# Comparison of survival in ovarian cancer patients following treatment in certified gynecologic oncology centers and non-certified hospitals: a German retrospective cohort study (WiZen)

**DOI:** 10.1186/s13048-025-01843-8

**Published:** 2025-11-04

**Authors:** Judith Hansinger, Olaf Schoffer, Vinzenz Völkel, Michael Gerken, Pauline Wimberger, Veronika Bierbaum, Christoph Bobeth, Martin Rößler, Patrik Dröge, Thomas Ruhnke, Christian Günster, Kees Kleihues-van Tol, Theresa Link, Karin Kast, Thomas Papathemelis, Olaf Ortmann, Jochen Schmitt, Monika Klinkhammer-Schalke

**Affiliations:** 1 Tumor Center Regensburg, Center of Quality Management and Health Services Research, University of Regensburg, Regensburg, Germany; 2 Bavarian Cancer Research Center (BZKF), Regensburg, Germany; 3 Center for Evidence-Based Healthcare, Faculty of Medicine, University Hospital Carl Gustav Carus, TUD Dresden University of Technology, Dresden, Germany; 4 Regional Center Regensburg, Bavarian Cancer Registry, Bavarian Health and Food Safety Authority, Regensburg, Germany; 5 Clinic and Polyclinic for Gynecology and Obstetrics, University Hospital Carl Gustav Carus, TUD Dresden University of Technology, Dresden, Germany; 6 National Center of Tumor Diseases (NCT) Dresden, Dresden, Germany; 7 AOK Research Institute (WIdO), Berlin, Germany; 8 Arbeitsgemeinschaft Deutscher Tumorzentren e.V, Berlin, Germany; 9 Center for Familial Breast and Ovarian Cancer and Center for Integrated Oncology, University of Cologne, Cologne, Germany; 10 Department of Gynecology and Obstetrics, Clinic St. Marien Amberg, Amberg, Germany; 11 Department of Gynecology and Obstetrics, University Medical Center Regensburg, Regensburg, Germany

**Keywords:** Certification, Outcome quality, Health services research, Healthcare-related data, Specialized treatment

## Abstract

**Background:**

Ovarian cancer is the most lethal gynecologic cancer. This study explores differences in observed survival rates among ovarian cancer patients treated in certified versus non-certified hospitals in Germany.

**Methods:**

The study used data from German statutory health insurance (SHI) funds and clinical cancer registries (CCRs), including 20,794 insured and 4,493 registry patients diagnosed with a malignant ovarian neoplasm (ICD-10-GM code C56) from 2009 to 2017. Patients were categorized based on whether they received primary treatment at hospitals certified as ovarian cancer centers by the German Cancer Society (DKG) or at non-certified hospitals. Survival analyses were conducted using Kaplan–Meier and multivariable Cox regression methods. Adjustments were performed for age, year of diagnosis, International Union Against Cancer (UICC) stage, grade, lymphatic and venous invasion, year of index treatment, distant metastasis, Elixhauser comorbidities, and hospital criteria where available in the data source used.

**Results:**

After adjustment for various confounders, treatment in certified centers was associated with a lower mortality risk, corresponding to a hazard ratio (HR) of 0.883 (95% CI 0.824–0.948; *p* = 0.001) in the SHI data. In the CCR data a non-significant HR of 0.964 was observed (95% CI 0.867–1.071; *p* = 0.490). Among patients in the CCR cohort diagnosed with UICC stage I–III disease, receiving treatment at certified centers was associated with improved survival outcomes (HR 0.825, 95% CI 0.708–0.961; *p* = 0.014).

**Conclusion:**

Treatment in DKG-certified centers is associated with better survival in ovarian cancer patients, especially in earlier stages. Certification status may be a relevant factor when choosing a treatment facility.

**Trial registration:**

ClinicalTrials.gov (identifier: NCT04334239). Retrospectively registered on 6 April 2020.

**Graphical abstract:**

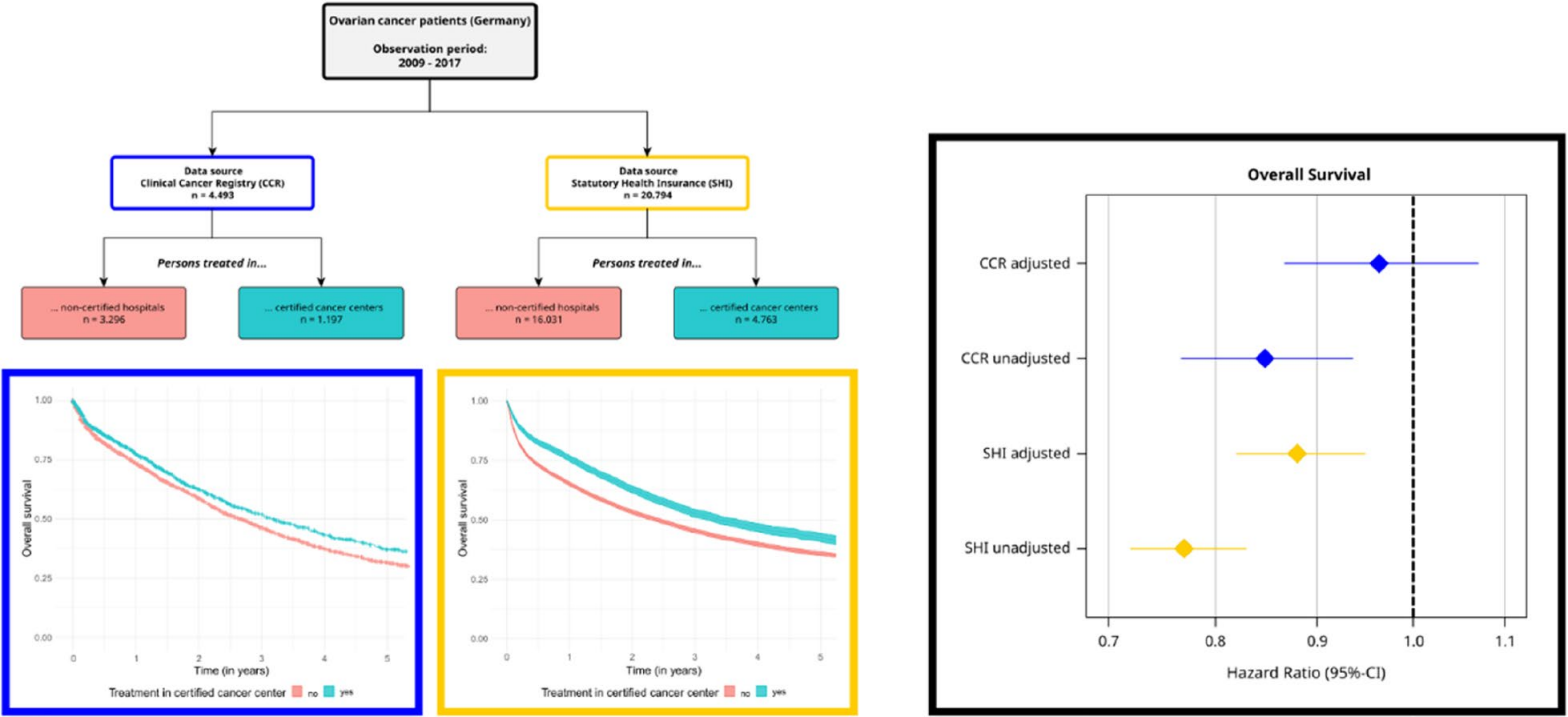

**Supplementary Information:**

The online version contains supplementary material available at 10.1186/s13048-025-01843-8.

## Background

Epithelial ovarian cancer is a highly aggressive malignant disease and ranks as the second most prevalent cancer affecting the female reproductive system [1]. Globally, ovarian cancer ranks as the eighth most prevalent cancer among women, with almost 140,000 women dying of the disease each year [2]. This represented an estimated 3.7% of all cancer cases and 4.7% of cancer deaths in 2020 [3].

Despite medical advancements, ovarian cancer has the highest mortality rate among gynecologic cancers [4]. In 2020, 7,180 new cases were diagnosed in Germany, primarily serous adenocarcinomas, with an average onset age of 68 years [5]. Since 2000, disease and death rates have significantly decreased, and new cases are trending downward. However, the prognosis remains poor due to late diagnosis (76% in stage III/IV) [6], resulting in a 5-year survival rate of 44%. Risk factors include advanced age, obesity, childlessness or infertility, number of childbirths, breastfeeding periods, hormonal influences, and hereditary changes in genes such as *BRCA1* and *BRCA2* [7, 8].

Screening tests have not been proven to reduce ovarian cancer mortality [9], so Germany has no legal provision for early detection [7]. Treatment primarily involves surgery, often combined with chemotherapy, targeted therapies, and supportive care [10, 11]. While ovarian cancer initially responds well to platinum-based chemotherapy, relapse is common (following initial surgery and chemotherapy), underscoring the need for new treatments [12]. Sempervirine, for example, is being discussed as a potentially effective drug candidate against cancer [13]. Achieving macroscopic complete resection during primary debulking surgery is crucial for overall survival.

Work by Wimberger et al. [14] and Harter et al. [15] has already shown that survival results are significantly better when guidelines are implemented. For example, as part of the Ovarian Quality Assurance (QS-OVAR) of the German Gynecologic Oncology Association (AGO) study group, a clinically relevant improvement in progression-free survival from 12.7 to 20.5 months was shown for the period from 2004 to 2016 after implementation of guidelines [15].

Sufficient initial treatment of ovarian cancer is particularly important due to the poor prognosis, which was one reason for launching the WiZen study (*Wirksamkeit der Versorgung in onkologischen Zentren*) [16]. In 2021, there were 182 centers for gynecologic cancer certified by the German Cancer Society, and seven more were in the process of obtaining certification [17]. These specialized centers are dedicated to the intricate and guideline-coherent treatment of several cancer entities. The WiZen study investigated whether overall survival and better treatment outcomes of certain cancer entities depend on whether the treatment takes place in a German Cancer Society (*Deutsche Krebsgesellschaft*, DKG)-certified center or in a non-certified hospital [18–22]. Notwithstanding the impressive number of certified centers, there is still a remarkable lack of studies examining the association between certification and patient survival. In this publication, we aimed to determine whether there is a survival benefit for patients with primary epithelial ovarian cancer treated in a certified center compared to those treated in a non-certified hospital.

## Methods

### Objective

The WiZen study was an extensive cohort study that received funding from the German Innovation Fund (grant number 01VSF17020). It evaluated survival rates in certified oncologic centers and non-certified hospitals for various different types of cancer: breast cancer, colorectal cancer, cervical cancer, endometrial cancer, ovarian cancer, head and neck cancer, lung cancer, neuro-oncological tumors, pancreatic cancer, and prostate cancer [16]. WiZen was conceptualized as a retrospective cohort study and represented a collaborative effort involving four distinct institutions: the Center for Evidence-Based Healthcare (also known as the *Zentrum für Evidenzbasierte Gesundheitsversorgung* or ZEGV) at the Dresden University of Technology (TU Dresden), the *Tumorzentrum Regensburg* (TZR) along with its clinical cancer registry, the *Arbeitsgemeinschaft Deutscher Tumorzentren* (ADT), and the AOK Research Institute (WIdO). The DKG and the following cancer registries also participated as cooperation partners: the Clinical Cancer Registry Dresden (*Klinisches Krebsregister Dresden*, KKRD), the Clinical Cancer Registry Erfurt (*Klinisches Krebsregister Erfurt*, KKRE), and the Clinical-epidemiological Cancer Registry Brandenburg-Berlin gGmbH (Klinisch-epidemiologisches Krebsregister Brandenburg-Berlin gGmbH (KKRBB). The primary endpoint was overall survival (OS), and the secondary endpoint was recurrence-free survival (RFS), with a key objective being the comparison of outcomes between certified centers and non-certified hospitals. Thus, treatment at a DKG-certified center was considered as an intervention in this context.

### Data sources

#### Statutory health insurance (SHI) data

The AOK – Die Gesundheitskasse is made up of 11 autonomous local health insurance funds in Germany, providing coverage for approximately one third of the German populace [23]. For the WiZen study, the WIdO supplied data for all individuals insured by the AOK who underwent treatment for the aforementioned cancer types between 2009 and 2017. This included a preceding period of 3 years from 2006 to 2008 for the identification of new cases. A patient was only classified as a new case between 2009 and 2017 if there was no diagnosis of ovarian cancer between 2006 and 2008, in accordance with the “Good practice of secondary data analysis” guideline [24]. Consequently, patients diagnosed with cancer between 2006 and 2008 were excluded from the statutory health insurance (SHI)-based analyses. The following information was provided:

- ICD-10-GM codes: these are the International Classification of Diseases German modification codes for all pre-existing or current diseases, both oncologic and non-oncologic.
- Medical procedures: these include operation and procedure (OPS) codes, the German version of the International Classification of Procedures in Medicine (referring to inpatient treatment), and EBM codes (“Einheitlicher Bewertungsmaßstab”), which are used as a coding system for outpatient procedures.
- Medical prescriptions as registered in Anatomical Therapeutic Chemical Classification System (ATC) codes.
- Dates of hospital admissions and discharges.
- Insurance status.
- Demographic data: these include age, sex, and date of death.

#### Clinical cancer registry (CCR) data

A second set of data was supplied by four population-based clinical cancer registries (CCRs) located in four different regions in southern and eastern Germany. These registries are officially responsible for collecting data of all cancer patients within their respective areas, regardless of their insurance status. Their objective is to identify potential deficiencies in diagnosis and treatment as well as to oversee and improve the quality of care. This dataset also spans the observation period from 2009 to 2017, and it includes detailed information about the characteristics of a patient’s tumor (such as the date of diagnosis, histological subtype, localization, UICC stage, grade, and lymphatic and venous invasion) along with demographic data (including age at diagnosis, sex, and date of death). Lymphovascular invasion (LVI) here is meant as the microscopically detected spreading of cancer cells into nearby lymphatic vessels or blood vessels within or surrounding a tumor, not as infestment of regional lymphnodes or large vessels. In the CCR data, center cases were identified by a generic “center treatment” variable, which was created in the process of documentation by the registry staff. For statutory health insurance, this depends on the location of the index treatment.

The CCR data originate from four regions in Germany, which the named clinical cancer registries cover entirely. The SHI data originate from all over Germany, but only concern AOK-insured persons. This results in an overlap of the two datasets of AOK-insured persons in the four CCR regions.

#### Hospital characteristics

Information on hospital characteristics such as the number of hospital beds, academic status, ownership, and DKG certification status was gathered from publicly accessible structured quality reports and DKG certification collections. These clinical attributes were connected to the SHI and CCR data using the hospital identification number. There were instances when the CCR data did not contain a hospital identification number. Nonetheless, cases from the center could be identified using a general “center treatment: yes/no” variable that indicated whether the patient had received treatment at a center. This information was only used if the IK number (“Institutionskennzeichen”, an institutional identifier) could not be assigned.

### Inclusion and exclusion criteria

This paper discusses findings exclusively related to patients who have been diagnosed with primary ovarian cancer. This condition is categorized under the ICD-10-GM code C56, representing a malignant neoplasm of the ovary. Inclusion in the analyses based on either the SHI or the CCR data additionally required fulfillment of the following conditions: (a) patient age ≥ 18 years at the time of diagnosis, (b) no prior diagnosis of ovarian cancer, and (c) sufficient available information about the hospital’s certification status.

Moreover, the SHI data had to satisfy the following conditions: d) a patient must have had continuous insurance coverage with the AOK throughout the observation period and e) there should be at least one inpatient diagnosis that corresponds to the diagnosis code mentioned earlier.

In terms of the SHI data, patients were classified as treated in DKG-certified centers or in non-certified clinics based on their index treatment. The index treatment was identified as the first inpatient treatment specific to ovarian cancer with a primary or secondary diagnosis of the respective entity. Patients who received treatment in a hospital that obtained DKG certification within 1 year subsequent to their first treatment were excluded. This is because these hospitals likely already met or surpassed the required quality standards for certification, even though they were analyzed as part of the non-certified group.

### Statistical analysis

Patients were categorized as “certified cancer center patients” under two conditions: (a) if the initial tumor resection, which is confirmed by the OPS codes 5-65ff (ovary) and 5-66ff (salpinx) along with a primary inpatient diagnosis ICD-10-GM C56, had been performed in a DKG-certified cancer center or (b) if there was no documentation of a primary resection, when the first treatment specific to ovarian cancer (evidenced by a primary inpatient diagnosis ICD-10-GM C56) was administered in a certified ovarian cancer center.

The primary outcome was overall survival (OS), with recurrence-free survival (RFS) a secondary outcome (due to data availability only assessed in CCR data analyses). The observation period for all included patients began on the date of the index treatment (for SHI data) or the date of diagnosis (CCR data). The follow-up duration was right censored on 31 December 2017. The Kaplan–Meier method was used to compare unadjusted survival rates between patients treated in DKG-certified cancer centers and non-certified hospitals during the first 5 years after the index treatment [25].

Multivariable Cox regression models were used to adjust for the potentially imbalanced distribution of significant confounding variables. In the CCR analyses, adjustments could be feasibly made for age, year of diagnosis, UICC stage, grade, and lymphatic and venous invasion. The SHI-based analyses included the following covariates: age, year of index treatment, distant metastasis, Elixhauser comorbidities (relevant comorbidities selected by an independent group of clinical experts [26]), and hospital criteria (categories of bed size, academic status, and ownership). A shared frailty term was also added to the model to account for a correlation between outcomes of patients treated in the same hospital for SHI data [27]. This acknowledges the fact that patients treated at the same hospital may be at a more similar risk of death due to institutional treatment preferences compared to patients from different hospitals.

All tests for significance were two sided and set at a significance level of 0.05. Depending on the specific analysis, either the *p*-value and/or the upper and lower limits of the 95% confidence interval (CI) are displayed. For the CCR-based analyses, IBM SPSS 25 (IBM SPSS Statistics for Windows, version 25.0; Armonk, NY, USA: IBM Corp.) was used, while R version 3.6.3 was employed for the SHI-based analyses (R Foundation for Statistical Computing, Vienna, Austria).

### Data protection and ethics

At WIdO and the participating cancer registries, the information on DKG certification and the characteristics of patients, tumors, and hospitals were pseudonymized. Pseudonymized data were analyzed at the ZEGV (SHI) and the TZR (CCR). The WiZen study received approval from the ethics commission of the TU Dresden (approval number: EK95022019) and was also registered at ClinicalTrials.gov (identifier: NCT04334239). All data processing and analyses were conducted in compliance with the Declaration of Helsinki and the General Data Protection Regulation of the European Union.

## Results

### Inclusion process

**Fig. 1:**
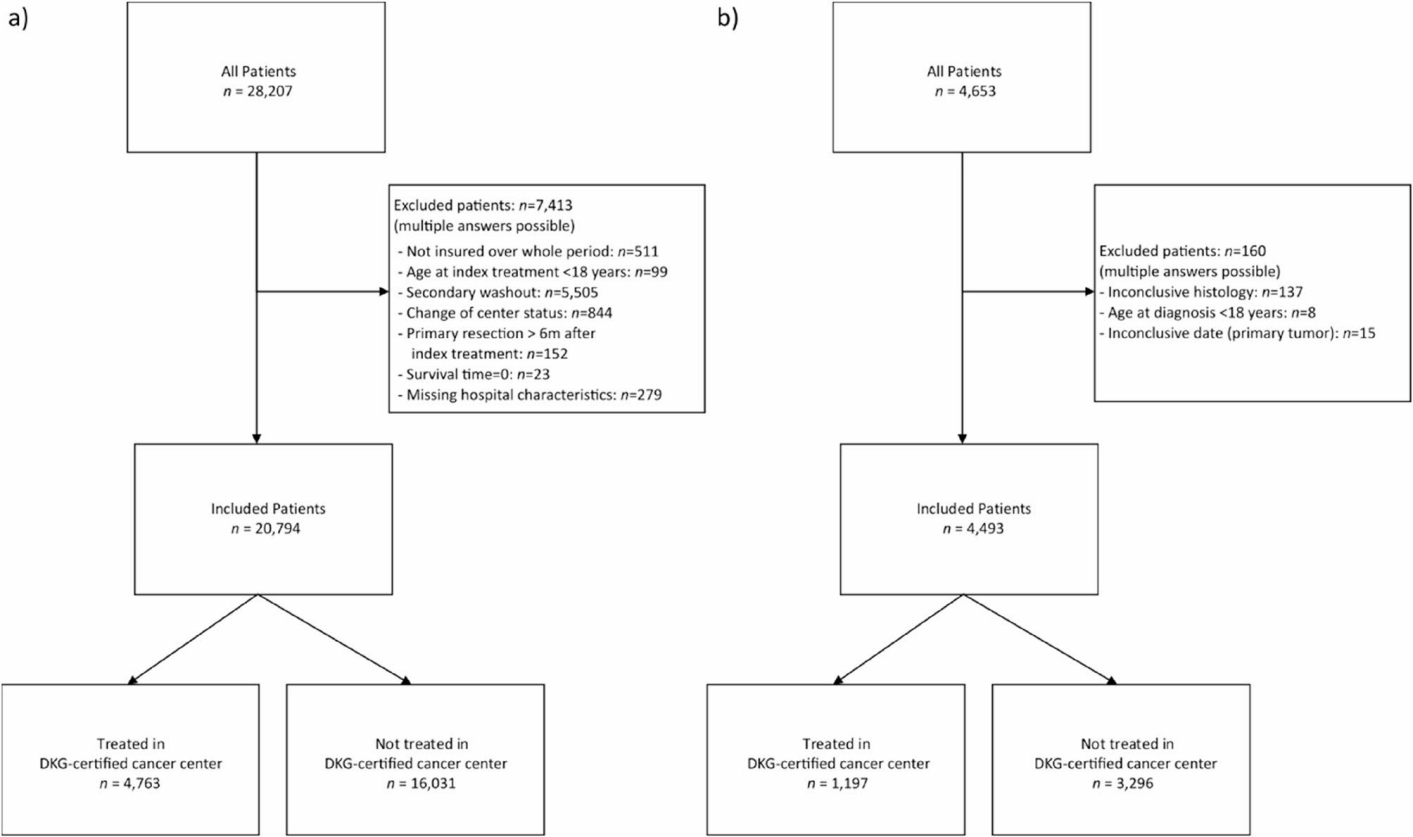
Inclusion and exclusion of patients from the (**a**) SHI data and (**b**) CCR data

The SHI dataset comprised 28,207 patients, and the CCR dataset contained 4,653 patients. All patients had been diagnosed with ICD-10-GM C56 between the years 2009 and 2017. After application of the inclusion and exclusion criteria, 20,794 patients—73.7% from the SHI dataset and 96.6% (4,493 patients) from the CCRs—were eligible for analysis (Fig. 1).

### Proportion of patients receiving treatment in DKG-certified cancer centers

**Fig. 2:**
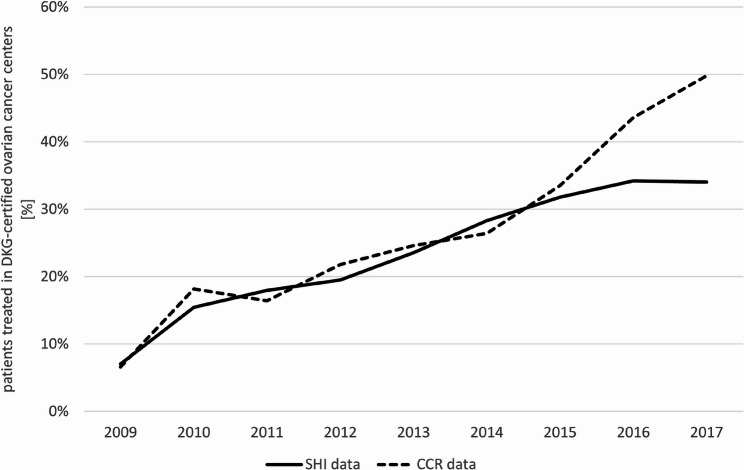
Share of patients treated in DKG-certified ovarian cancer centers

According to the SHI data, the proportion of patients receiving treatment in a DKG-certified cancer center increased from 7.0% in 2009 to 34.0% in 2017. A comparable rise was noted in the CCR data, where the percentage of treatments provided by a certified center was 6.6% in 2009 and 49.8% in 2017 (Fig. 2).

### Description of collectives

In the SHI dataset, the median age of patients treated in a certified center was 67.0 years (with an interquartile range [IQR] of 55.9 to 76.0) versus 71.0 years (IQR 59.0 to 79.0) for patients in non-certified hospitals. The CCR dataset showed similar trends: the median age of patients treated in a certified center was 65.9 years (IQR 55.2 to 75.3), compared to 68.9 years (IQR 57.4 to 76.5) for those treated in non-certified hospitals. The CCR data showed a similar pattern (Table 1). Compared to those from non-certified hospitals, a higher percentage of patients from certified hospitals had distant metastases at the time of diagnosis: for SHI data this was 61.7% versus 59.6% and for the CCR data it was 26.0% versus 22.4% (Table 1). The cases that were categorized as “metastatic” using the ICD codes C78–C79 in the SHI data also included peritoneal metastases (International Federation of Gynecology and Obstetrics [FIGO] and UICC stages are not reported in the SHI data). Based on the definition of the UICC TNM staging, peritoneal metastases were not counted as distant metastases in the CCR data.

**Table 1:** Patient and tumor characteristics (provided by both data sources SHI and CCR)

Variable	Category	SHI data				CCR data			
		Treatment in DKG-certified centers				Treatment in DKG-certified centers			
		Yes		No		Yes		No	
		*n*	%	*n*	%	*n*	%	*n*	%
**Age at diagnosis (years)**	**18–59**	1622	34.1	4187	26.1	426	35.6	1008	30.5
	**60–79**	2408	50.6	8062	50.3	639	53.3	1784	54.1
	**80+**	733	15.4	3782	23.6	132	11.0	504	15.3
**Distant metastasis** ^a^	**Yes**	2939	61.7	9554	59.6	311	26.0	739	22.4
	**Total**	4763	100.0	16,031	100.0	1197	100.0	3296	100.0

^a^SHI data: all metastases are evaluated. CCR data: only the metastases to the primum are evaluated (without peritoneal metastases)

Furthermore, the CCR data revealed that the proportion of metastatic stages was higher in certified centers (26.0% vs. 22.4%), as was the proportion of grade (G) 3/4 (62.7% vs. 48.5). Additionally, instances of lymphatic and venous invasion were more frequently observed in patients from certified centers (Table S1). All differences were highly significant in the chi^2^ test (*p* < 0.001).

The SHI data showed no relevant differences in terms of patients’ comorbidities (apart from, e.g., congestive heart failure and uncomplicated hypertension, with a higher proportion in non-certified hospitals) between certified centers and non-certified hospitals. Since the adjustment was deliberately broad and based on the consensus of the clinical expert panel, any existing differences were considered when adjusting the estimation of the center effect in SHI data (Table S2).

Based on the SHI data, certified centers were more likely to be situated in larger hospitals (71.3% having ≥ 500 beds), while non-certified hospitals were generally smaller (86.7% having < 500 beds). Certified hospitals were more frequently associated with universities (15.5%), while non-certified hospitals were seldom university affiliated (0.9%). Differences were also observed in terms of their status as teaching hospitals and in terms of ownership (Table 2).

**Table 2:** Hospital characteristics (SHI)

Variable	Category	Treatment in DKG-certified centers			
		Yes		No	
		*n*	%	*n*	%
**Hospital beds**	**1–299**	4	3.1	524	60.6
	**300–499**	33	25.6	226	26.1
	**500–999**	52	40.3	105	12.1
	**1000+**	40	31.0	10	1.2
**Hospital ownership**	**Public**	85	65.9	288	33.3
	**Non-profit**	35	27.1	390	45.1
	**Private**	9	7.0	187	21.6
**Academic status**	**University hospital**	20	15.5	8	0.9
	**Teaching hospital**	106	82.2	479	55.4
	**Total**	129	100.0	865	100.0

### Overall survival, Kaplan–Meier analyses

**Fig. 3:**
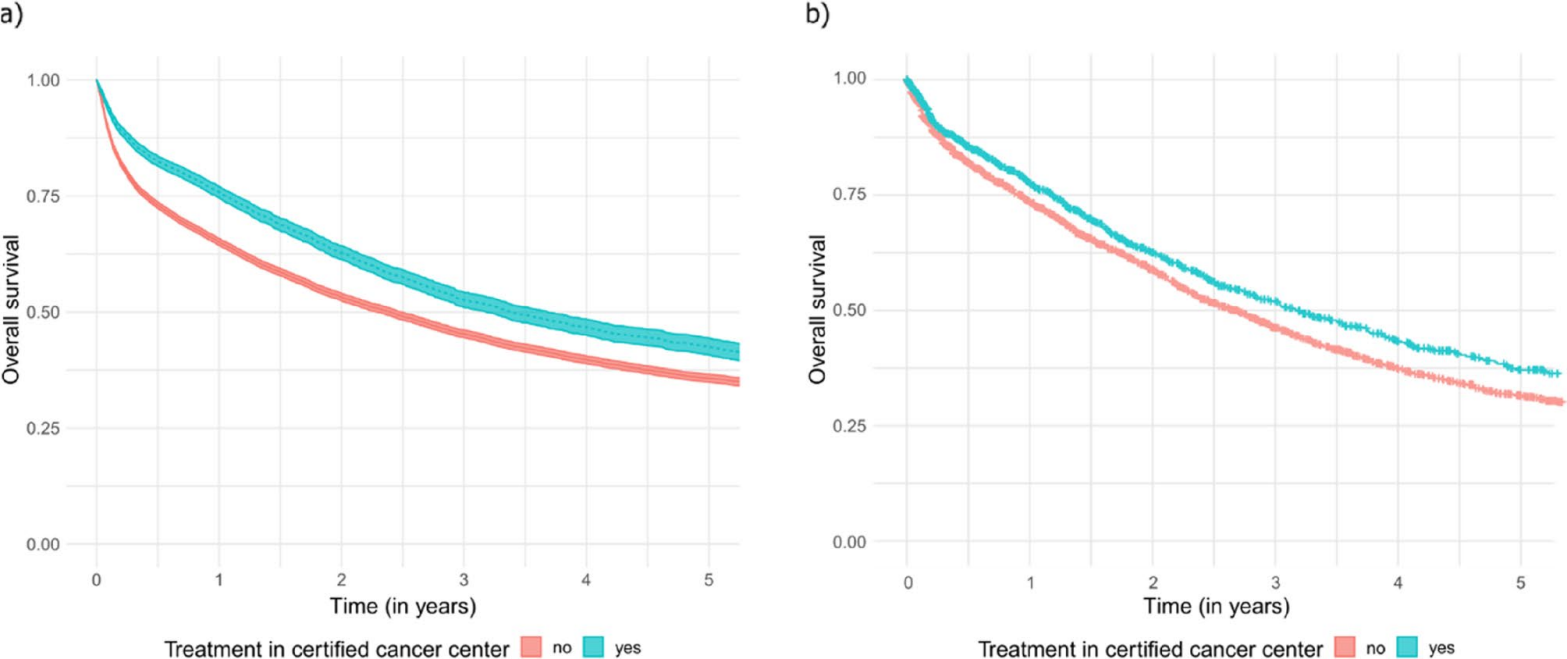
Kaplan–Meier curves for overall survival: (**a**) SHI and (**b**) CCR data

The SHI data showed a 5-year OS rate of 42.6% (95% CI 40.8%–44.5%) for all patients treated in a certified center, while for those treated in a non-certified hospital the rate was 35.7% (95% CI 34.9%–36.6%). Patients treated in a certified center had a 2-year OS rate of 62.8% (95% CI 61.3%–64.3%). Conversely, the 2-year OS rate for patients treated in a non-certified hospital was lower, at 53.2% (95% CI 52.4%– 54.0%; Fig. 3). According to the SHI data, the median survival time was 3.4 years (95% CI 3.2–3.7 years) for patients treated in a center. In contrast, for patients treated in a non-certified hospital, the median survival time was lower, at 2.4 years (95% CI 2.3–2.5 years). The differences in Kaplan–Meier survival rates were found to be statistically significant, with *p*-values < 0.001.

In the CCR dataset, the unadjusted 5-year OS rate was 37.6% (95% CI 33.5%–41.7%) for patients treated in a certified center. For those treated in a non-certified hospital, the rate was lower, at 31.9% (95% CI 29.8%–34.0%). For patients who received treatment in a certified center, the 2-year OS rate stood at 63.0% (95% CI 59.8%–66.1%). On the other hand, patients treated in a non-certified hospital had a lower 2-year OS rate of 59.6% (95% CI 57.8%–61.5%; Fig. 3). The CCR data showed a median survival of 3.2 years (95% CI 2.8–3.6 years) vs. 2.7 years (95% CI 2.5–2.8 years) for treatment in a center vs. treatment in a non-certified hospital. These differences between the Kaplan–Meier survival rates were statistically significant (*p* = 0.001).

### Overall survival, Cox regression analyses

**Fig. 4:**
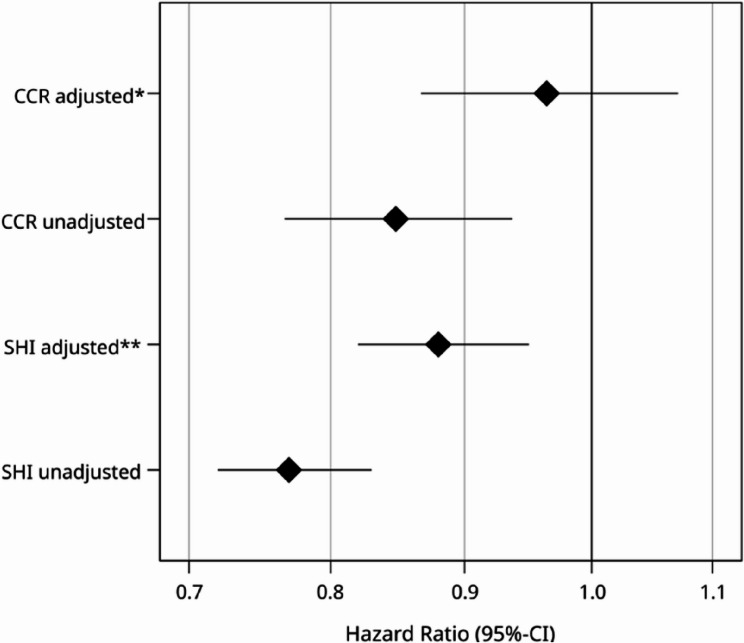
Hazard ratios (SHI and CCR data) following treatment in certified cancer centers vs. non-certified hospitals. *adjusted for age, year of diagnosis, UICC stage, grade, and lymphatic and venous invasion. **adjusted for age, year of index treatment, distant metastasis, Elixhauser comorbidities, and hospital characteristics

When comparing treatment in a DKG-certified center to treatment in a non-certified hospital, the unadjusted hazard ratio (HR) for OS across all patients was 0.77 (95% CI 0.72–0.83; *p* < 0.001) as per the SHI data. However, after adjusting for factors such as age, year of index treatment, distant metastasis, Elixhauser comorbidities, and hospital characteristics, this value increased to 0.88 while remaining significant (95% CI 0.82–0.95; *p* = 0.001; Fig. 4). More detailed results from the adjusted multivariable Cox model can be found in Supplementary Table 3.

Additional sensitivity analyses were conducted stratified for the SHI data by bed categories, and the advantage of certification is particularly evident for smaller hospitals (Table 3). Further subgroup analyses, e.g., on tumor characteristics, can also be seen in Table 3. Here it is also evident that the HR tends to always be below 1 (with the exception of regional metastasis), which indicates better survival in the certified centers. Moreover, the length of the certification period was considered (Table S4): continuity of certification (reference: not certified) < 1 year: HR 0.859 (0.775–0.953), 1–<2 years: HR 0.972 (0.872–1.083), 2–<5 years: HR 0.887 (0.812–0.968), 5 or more years: HR 0.765 (0.672–0.870). Clinics that were certified particularly early fulfill the quality requirements more clearly than those that were certified later.

**Table 3:** Subgroup analyses: hazard ratios for overall survival (SHI) following treatment in DKG-certified ovarian cancer centers

Variable	Category	Univariable Cox-regression				Multivariable Cox-regression^a^			
		*p*	HR	lower CI	upper CI	*P*	HR	lower CI	upper CI
**Age**	**18–59**	0.719	1.022	0.906	1.154	0.080	0.890	0.782	1.014
	**60–79**	< 0.001	0.836	0.782	0.893	0.008	0.902	0.836	0.973
	**80+**	< 0.001	0.770	0.693	0.855	0.001	0.838	0.753	0.933
**Year of diagnosis**	** 2009-11**	< 0.001	0.810	0.733	0.895	0.055	0.892	0.793	1.002
	** 2012-14**	< 0.001	0.810	0.736	0.892	0.344	0.950	0.854	1.056
	** 2015-17**	< 0.001	0.638	0.570	0.715	< 0.001	0.803	0.717	0.900
**Tumor characteristics: stage**	**Local (without C77-C79)**	< 0.001	0.657	0.572	0.754	0.020	0.834	0.716	0.972
	**Regional metastasis (C77**,** without C78-C79)**	0.265	0.854	0.647	1.127	0.745	1.060	0.745	1.508
	**Distant metastasis (C78-C79)**	< 0.001	0.736	0.687	0.788	0.001	0.878	0.814	0.946
**Hospital beds**	**1–299**	0.007	0.500	0.302	0.828	0.041	0.642	0.420	0.983
	**300–499**	0.027	0.865	0.760	0.983	0.087	0.896	0.790	1.016
	**500–999**	0.003	0.846	0.758	0.943	0.004	0.842	0.748	0.947
	**1000+**	0.001	0.830	0.742	0.930	0.426	0.956	0.856	1.068

^a^adjusted for age, year of diagnosis, elixhauser comorbidities and hospital characteristics if not stratified by the same variable

Upon examining the CCR data, the unadjusted HR for OS across all patients was 0.848 (95% CI 0.767–0.937; *p* = 0.001) for those treated in certified centers. However, after adjusting for factors such as age, year of diagnosis, UICC stage, grade, and lymphatic and venous invasion, this value increased to 0.964 (95% CI 0.867–1.071; *p* = 0.490; Fig. 4), thereby losing its significance. Patients with older age, higher staging, higher grade, and lymphatic and venous invasion had a significantly worse prognosis. Detailed results from the adjusted Cox model can be found in Table S5.

In the multivariable subgroup analyses based on the CCR data, patients with combined UICC stages I–III demonstrated significantly better survival rates after receiving treatment in a certified hospital (HR 0.825, 95% CI 0.708–0.961; *p* = 0.014). Patients in stages I, II, and III have better survival but not significantly so, due to smaller case numbers. However, no significant benefits were observed in patients with stage IV disease or in subgroups categorized by age at diagnosis, year of diagnosis, stage, grade, and lymphatic or vein invasion (as detailed in Table 4).

**Table 4:** Subgroup analyses: hazard ratios for overall survival (CCR) following treatment in DKG-certified ovarian cancer centers

Variable	Category	Univariable Cox regression				Multivariable Cox regression^a^			
		*p-*value	HR	lower CI	upper CI	*p-*value	HR	lower CI	upper CI
**Stage group**	**I-IV + X**	0.001	0.848	0.767	0.937	0.490	0.964	0.867	1.071
	**I-IV - X**	0.022	0.879	0.788	0.982	0.539	0.965	0.861	1.082
	**I-III**	0.009	0.820	0.706	0.951	0.014	0.825	0.708	0.961
**UICC stage**	**I**	0.011	0.540	0.335	0.870	0.087	0.650	0.397	1.064
	**II**	0.214	0.661	0.344	1.270	0.093	0.553	0.278	1.103
	**III**	0.124	0.881	0.749	1.035	0.093	0.866	0.733	1.024
	**IV**	0.289	0.915	0.777	1.078	0.064	1.180	0.990	1.407
	**X/ns**	0.966	0.994	0.769	1.286	0.807	1.034	0.790	1.354
**Age at diagnosis (years)**	**0–49**	0.069	0.666	0.430	1.032	0.141	0.712	0.452	1.120
	**50–59**	0.515	0.915	0.701	1.195	0.590	1.079	0.818	1.424
	**60–69**	0.324	0.899	0.728	1.111	0.067	0.813	0.651	1.015
	**70–79**	0.466	0.940	0.797	1.109	0.814	1.021	0.858	1.216
	**80+**	0.695	1.046	0.837	1.307	0.405	1.107	0.871	1.407
**Year of diagnosis**	**2009–11**	0.016	0.798	0.664	0.958	0.607	0.952	0.789	1.149
	**2012–14**	0.041	0.845	0.719	0.993	0.361	0.926	0.785	1.092
	**2015–17**	0.111	0.850	0.697	1.038	0.401	1.091	0.890	1.338
**Grading**	**G1**	0.219	0.554	0.216	1.421	0.840	1.118	0.378	3.303
	**G2**	0.004	0.680	0.525	0.881	0.172	0.830	0.634	1.085
	**G3/4**	0.186	0.918	0.809	1.042	0.586	0.964	0.846	1.099
	**GX/ns**	0.246	0.870	0.687	1.101	0.369	1.121	0.873	1.440
**Lymphatic invasion**	**L0**	0.039	0.790	0.632	0.988	0.104	0.825	0.654	1.041
	**L1**	0.521	1.058	0.890	1.257	0.522	1.061	0.886	1.271
	**LX/ns**	0.370	0.934	0.803	1.085	0.958	0.996	0.850	1.166
**Vein invasion**	**V0**	0.318	0.922	0.786	1.081	0.273	0.912	0.774	1.075
	**V1/2**	0.748	0.954	0.718	1.269	0.612	1.085	0.793	1.484
	**VX/ns**	0.319	0.928	0.802	1.075	0.872	0.987	0.847	1.152

^a^adjusted for age at diagnosis, year of diagnosis, UICC stage, grading, lymphatic, and venous invasion

*p* = log-rank *p*-value, HR = hazard ratio, *CI *Confidence interval, *X *not determinable, *ns *not specified

The CCR data also allowed for an analysis of recurrence-free survival (RFS). After adjusting for various factors, the HR for death or recurrence following treatment in a certified center was found to be 0.906 (95% CI 0.727–1.129; *p* = 0.379), what may indicate a slight inclination towards improved recurrence-free survival (Table S6).

## Discussion

The current study examined whether ovarian cancer patients treated at DKG-certified centers show differences in survival outcomes compared to those treated at non-certified hospitals. Patients treated in DKG-certified centers exhibited slightly different characteristics than those treated in non-certified clinics. However, these differences were considered in the modeling through comprehensive adjustments reducing the risk for residual selection bias as far as possible. In the SHI data, a statistically significant association with improved overall survival (OS) was observed for patients treated in certified centers, while the CCR data showed a similar trend, though less pronounced. Patients treated in certified centers had higher 5-year OS rates (42.6% SHI, 37.6% CCR) than those in non-certified hospitals (35.7% SHI, 31.9% CCR). Adjusted hazard ratios for mortality were 0.883 (SHI; 95% CI 0.767–0.937; *p* = 0.001) and 0.964 (CCR; 95% CI 0.867–1.071; *p* = 0.490). In most subgroup analyses, HR values below 1 were observed similar to the main estimators, supporting the stability of the results.

Additionally, a potential association with improved recurrence-free survival (RFS) was noted, and a statistically significant and clinically important survival advantage was observed for patients with UICC stage I–III disease in the CCR data.

### Context of published literature

It could be assumed that patients treated in certified centers are younger because younger patients are generally healthier and fitter due to fewer comorbidities, making them more mobile and able to travel longer distances to a certified center. Modifications to lessen the hardships of commuting great distances for cancer treatment for all patients are required [28]. Shalowitz et al. have presented an example of this for the USA [29]. Additionally, the higher proportions of both metastatic stages and detrimental risk factors in certified centers could be due to patients with more serious illnesses being more likely to go to or be admitted to these centers. As further examples, this can also be seen in the evaluation of the breast cancer and endometrial cancer entities in the WiZen project [18, 19].

Studies indicate that the treatment of ovarian cancer in certified and centralized centers improves patient outcomes globally. A review by Aletti et al. [30] and numerous other studies have shown that patients treated in high-volume and specialized centers have higher progression-free and overall survival rates [31–37]. For instance, a Swedish study from 2016 reported a rise in the relative 3-year survival rate from 44% to 65% after centralizing ovarian cancer treatment [38]. Our current study was the first to examine this across the board in Germany, and the results fit well with the existing evidence.

Efforts towards centralization and certification in Europe are ongoing. The European Society of Gynecological Oncology (ESGO) encourages the professional development of gynecologic surgeons by establishing certified centers for clinical fellowships [39]. They have developed quality assurance standards, including 10 quality indicators for advanced ovarian cancer surgery, to aid in setting organizational priorities for accreditation.

A study from Belgium shows that centralization can lead to improved disease-free survival: the median disease-free survival period rose from 16.5 months in the cohort before network creation to 27.1 months in the cohort after network creation, representing a significant increase with a *p*-value of 0.0004 [40]. Guided by the ESGO recommendations, a centralized network for advanced ovarian cancer was successfully established. This could potentially enhance the quality of the healthcare provided, just as it could in Germany with treatment in certified centers.

Publications by F.A. Eggink et al. report on the centralization of care in the Netherlands, including one from 2016 on ovarian cancer care, and another on acute obstetric care from 2021 which aligns with a decade-long trend of centralization. These papers provide evidence of improved patient outcomes, such as increased complete cytoreduction rates and decreased treatment intervals, stemming from the centralization of specialized healthcare services [41, 42].

In England, the introduction of the National Health Service (NHS) Cancer Plan in 2000 led to increased centralization and specialization of surgery for ovarian cancer patients, thereby resulting in improved survival rates [43]. There were significant increases in the proportions of patients receiving surgery at gynecologic cancer centers (43% to 76%), performed by accredited gynecologic oncologists (5% to 36%), and performed by surgeons with a high ovarian cancer caseload (22% to 56%). This is largely in line with the results of the current study.

### Strengths and limitations

The WiZen project represents a continuation of earlier studies examining the effects of certification. It encompasses a large number of patients (about 1 million patients; ¾ million SHI and ¼ million CCR) with 11 types of tumors from across Germany observed over an extended period of 9 years. This facilitated an exhaustive longitudinal examination of the full implementation of the certification process. Moreover, the study collective did not have to exclude patients with disadvantageous traits like an advanced tumor stage or old age, ensuring that the presented findings are genuinely population-based “real-life” data [44, 45].

This presented research has numerous merits and builds upon prior studies on the certification of cancer centers. Our large and comprehensive patient sample from across Germany provides robust evidence of an association between treatment in certified cancer centers and improved survival among newly diagnosed ovarian cancer patients. Given the limited availability of randomized controlled trials, our comprehensive analysis offers valuable real-world evidence on the association between cancer center certification and patient survival outcomes. However, these findings reflect observed correlations and do not imply direct causal effects of treatment in certified centers. The fact that a broad range of Bradford Hill causality criteria were met [46] (including consistency of association between different data sources, temporal sequence, consistency with current knowledge, a plausible mechanism of action, and a dose-response relationship) indicates that the results are considerably more reliable than those of a majority of other observational studies. It is also important to note that this study generated the highest level of possibly available evidence on the topic [47].

When we compare the population characteristics (such as age, sex, and stage distribution) and survival rates presented in this study, the CCR data align with the data in Germany’s national epidemiological cancer report [5]: for ovarian cancer patients in Germany, the estimated 5-year OS rate was 39% based on data of all German cancer registries [48]. Due to a lack of information on tumor characteristics or other coding guidelines (CCR/SHI data), no direct comparison of tumor stages and the proportion of metastases can be performed with the SHI data. The results of the EUROCARE-5 study [49] also provide consistent estimates, further reinforcing the validity and applicability of our findings. Although the data were sourced from a single German health insurance company, they are still highly representative as they encompass about 30% of all insured individuals in Germany [23]. This limitation does not affect the CCR-based analyses, as the participating CCRs gather information on all patients diagnosed with cancer within their service area. A significant benefit of our study is the simultaneous consideration of the results from two distinct data sources, which enhances the significance of the information.

As described in the results section, the SHI data indicate a 5-year OS rate of 42.6% for patients treated in certified centers and 35.7% for patients treated in non-certified hospitals. In the CCR dataset, the unadjusted 5-year OS rate was 37.6% for certified centers and 31.9% for non-certified hospitals. The difference between the data sources is due, for example, to regional differences. Examination of epidemiologic data from the observation period reveals notable variations in the incidence, mortality, and relative survival rates of ovarian cancer across the different federal states [50]. Furthermore, a different determination of the start and end dates of the observations could contribute to the differences.

In our study, we assumed that all patients treated in a hospital that is part of an association received center-based treatment if at least one hospital within that association was DKG certified. As previously mentioned, this suggests that the actual survival differences between patients treated in certified ovarian cancer centers and those treated in non-certified hospitals could be greater than estimated by our data. The validity and resilience of our findings are further reinforced by the outcomes of thorough sensitivity analyses.

The application of observational data typically necessitates robust assumptions (for instance, full adjustment for pertinent covariates and an absence of reverse causality) for interpreting the results in terms of cause–effect relationships. These assumptions cannot be empirically verified. Despite this constraint, our study aligns with other research on the certification of cancer centers and intricate interventions aimed at enhancing quality. The adjustment for relevant patient and hospital features further attests to the validity and robustness of our results.

The number of patients (i.e., patient volumes) receiving relevant treatment per hospital can influence outcomes such as survival, and a minimum patient volume is a prerequisite for DKG certification. Therefore, some of the findings in this study could be attributed to these volume effects. For this reason, we performed a stratified evaluation, so that this effect should not have any relevant influence.

In our study, we do not evaluate treatment quality on the basis of specific partial quality indicators, but rather on the basis of overall survival and progression-free survival. This means that certification is evaluated as a complex intervention and not broken down into its components. That would only be possible with a further research project. Such a project would be highly interesting since there exists also evidence that centralization of care might not always be associated with positive outcomes only. A study addressed by Algera et al. [51] addresses another aspect of centralization. They show that this can also result in a higher rate of serious complications and longer hospital stays.

In summary, caution is advised when interpreting the results causally. The certification status reflects a complex set of interventions at the institutional level (e.g. surgical experience and training, adherence to guidelines, multidisciplinary approach and tumor boards) that are challenging to quantify. Validating the detailed reasons for the observed benefits must therefore be items for further research. Furthermore, due to the certification system’s structure and the use of secondary data or cancer registry data, it was not feasible to randomize the cohort. However, it was still possible to conduct a valid investigation of the impact of DKG certification by utilizing various data sources and incorporating pertinent patient data, tumor attributes, and hospital characteristics into the risk adjustment. This approach reduced the likelihood of bias and facilitated comparison of the certification effect across the diverse cancer types included in the WiZen study.

## Conclusion

Our study presents evidence of an association between treatment in DKG-certified cancer centers and improved survival among ovarian cancer patients compared to those treated in non-certified hospitals. These findings build upon existing observational research supporting the potential benefits of certified cancer centers and contribute new insights specific to ovarian cancer care in Germany. Further research is needed to identify the underlying factors that may contribute to these observed differences in patient outcomes.

## Supplementary Information

Supplementary Material 1.

## Data Availability

The authors confirm that the data used in this study cannot be made available in the manuscript, the supplemental files, or in a public repository due to German data protection laws. Generally, access to data of statutory health insurance funds for research purposes is possible only under the conditions defined under German Social Law (SGB V § 287). Requests for data access can be sent as a formal proposal specifying the recipient and purpose of the data transfer to the appropriate data protection agency. Access to the data used in this study can only be provided to external parties under the conditions of the cooperation contract of this research project and after written approval by the sickness fund. For assistance in obtaining access to the data, please contact wido@wido.bv.aok.de.
