# Supplementary material for: Comparison of survival in ovarian cancer patients following treatment in certified gynecologic oncology centers and non-certified hospitals: a German retrospective cohort study (WiZen)
Source: J Ovarian Res. 2025 Nov 4;18:238. doi: 10.1186/s13048-025-01843-8 (PMC12587621; doi:10.1186/s13048-025-01843-8)
Supplement: Supplementary file 1 — Supplementary Material 1. [file 13048_2025_1843_MOESM1_ESM.docx]

Supplementary Material

**Comparison of survival in ovarian cancer patients following treatment in certified gynecologic oncology centers and non-certified hospitals: a German retrospective cohort study (WiZen)**

BMC Journal of Ovarian Research

Judith Hansinger

Tumor Center Regensburg, Center of Quality Management and Health Services Research, University of Regensburg, Regensburg, Germany

Bavarian Cancer Research Center (BZKF), Regensburg, Germany

[judith.hansinger@klinik.uni-regensburg.de](mailto:judith.hansinger@klinik.uni-regensburg.de)

**Table S1** Tumor characteristics according to treatment in DKG-certified centers yes vs. no (CCR data)

|  | **Treatment in DKG-certified centers** | **Yes** | | **No** | |
| --- | --- | --- | --- | --- | --- |
|  | | *n* | % | *n* | % |
| **UICC stage** | **I/0** | 212 | 17.7 | 612 | 18.6 |
|  | **II** | 77 | 6.4 | 188 | 5.7 |
|  | **III** | 465 | 38.8 | 1178 | 35.7 |
|  | **IV** | 311 | 26.0 | 739 | 22.4 |
|  | **X** | 132 | 11.0 | 579 | 17.6 |
| **Grade** | **G1** | 54 | 4.5 | 188 | 5.7 |
|  | **G2** | 219 | 18.3 | 765 | 23.2 |
|  | **G3/4** | 750 | 62.7 | 1597 | 48.5 |
|  | **GX** | 174 | 14.5 | 746 | 22.6 |
| **Lymphatic invasion** | **L0** | 446 | 37.3 | 1096 | 33.3 |
|  | **L1** | 382 | 31.9 | 807 | 24.5 |
|  | **LX** | 369 | 30.8 | 1393 | 42.3 |
| **Venous invasion** | **V0** | 658 | 55.0 | 1531 | 46.5 |
|  | **V1/2** | 150 | 12.5 | 351 | 10.6 |
|  | **VX** | 389 | 32.5 | 1414 | 42.9 |
|  | **total** | 1197 | 100.0 | 3296 | 100.0 |

**Table S2** Distribution of Elixhauser comorbidities (SHI data)

| **Treatment in DKG-certified centers** | **Yes** | | **No** | |
| --- | --- | --- | --- | --- |
|  | ***n*** | **%** | ***n*** | **%** |
| **Other oncological disease** | 1986 | 41.7 | 6315 | 39.4 |
| **Congestive heart failure** | 1071 | 22.5 | 4705 | 29.3 |
| **Heart rhythm disorders** | 1474 | 30.9 | 5071 | 31.6 |
| **Heart valves disease** | 761 | 16.0 | 2537 | 15.8 |
| **Pulmonary circulation disorders** | 373 | 7.8 | 1179 | 7.4 |
| **Peripheral vascular disorders** | 1078 | 22.6 | 3560 | 22.2 |
| **Hypertonus (uc)** | 3270 | 68.7 | 11874 | 74.1 |
| **Hypertonus (c)** | 945 | 19.8 | 3562 | 22.2 |
| **Neurodegenerative disorders** | 369 | 7.7 | 1447 | 9.0 |
| **Chronic pulmonary disease** | 2205 | 46.3 | 6565 | 41.0 |
| **Diabetes (uc)** | 1187 | 24.9 | 4793 | 29.9 |
| **Diabetes (c)** | 597 | 12.5 | 2422 | 15.1 |
| **Renal failure** | 810 | 17.0 | 3117 | 19.4 |
| **Liver disease** | 1227 | 25.8 | 3839 | 23.9 |
| **Obesity** | 1554 | 32.6 | 5061 | 31.6 |
| **Blood loss anemia** | 161 | 3.4 | 610 | 3.8 |
| **Deficiency anemia** | 779 | 16.4 | 2491 | 15.5 |
| **Alcohol abuse** | 158 | 3.3 | 501 | 3.1 |
| **Drug abuse** | 100 | 2.1 | 319 | 2.0 |

c: complicated, uc: uncomplicated

**Table S3** Full results for overall survival from adjusted multivariable Cox regression with shared frailty (SHI data)

|  |  | *p*-value | HR | lower 95%CI | upper 95%CI |
| --- | --- | --- | --- | --- | --- |
| Treatment in center | **no** |  | ref. |  |  |
|  | **yes** | 0.001 | 0.883 | 0.824 | 0.948 |
| Age (years) | **18–59** |  | ref. |  |  |
|  | **60–79** | <0.001 | 1.846 | 1.741 | 1.957 |
|  | **80+** | <0.001 | 3.936 | 3.674 | 4.216 |
| Distant metastasis | **yes** | <0.001 | 3.463 | 3.308 | 3.625 |
| Other oncological disease | **yes** | 0.796 | 0.995 | 0.958 | 1.034 |
| Congestive heart failure | **yes** | <0.001 | 1.341 | 1.280 | 1.405 |
| Cardiac arrhythmias | **yes** | 0.338 | 1.021 | 0.979 | 1.064 |
| Pulmonary circulation disorders | **yes** | <0.001 | 1.381 | 1.293 | 1.474 |
| Valvular disease | **yes** | 0.630 | 0.987 | 0.938 | 1.040 |
| Peripheral vascular disorders | **yes** | 0.052 | 1.045 | 1.000 | 1.093 |
| Hypertension (uc) | **yes** | 0.019 | 1.067 | 1.011 | 1.126 |
| Hypertension (c) | **yes** | 0.001 | 0.921 | 0.877 | 0.967 |
| Other neurological disorders | **yes** | <0.001 | 1.355 | 1.276 | 1.440 |
| Chronic pulmonary disease | **yes** | <0.001 | 0.928 | 0.893 | 0.964 |
| Diabetes (uc) | **yes** | <0.001 | 1.155 | 1.099 | 1.214 |
| Diabetes (c) | **yes** | 0.998 | 1.000 | 0.940 | 1.063 |
| Renal failure | **yes** | <0.001 | 1.364 | 1.299 | 1.431 |
| Liver disease | **yes** | 0.139 | 0.967 | 0.925 | 1.011 |
| Obesity | **yes** | <0.001 | 0.922 | 0.883 | 0.962 |
| Blood loss anemia | **yes** | 0.530 | 0.969 | 0.879 | 1.069 |
| Deficiency anemia | **yes** | <0.001 | 1.117 | 1.061 | 1.177 |
| Alcohol abuse | **yes** | <0.001 | 1.331 | 1.195 | 1.481 |
| Drug abuse | **yes** | 0.240 | 0.923 | 0.809 | 1.055 |
| Hospital beds | **1–299** |  | ref. |  |  |
|  | **300–499** | 0.045 | 0.938 | 0.881 | 0.999 |
|  | **500–999** | 0.002 | 0.890 | 0.828 | 0.957 |
|  | **1000+** | 0.007 | 0.867 | 0.781 | 0.961 |
| Teaching hospital | **yes** | 0.976 | 0.999 | 0.944 | 1.057 |
| University hospital | **yes** | 0.406 | 0.948 | 0.835 | 1.076 |
| Hospital ownership | **public** |  | ref. |  |  |
|  | **non-profit** | 0.334 | 0.973 | 0.922 | 1.028 |
|  | **private** | 0.713 | 0.987 | 0.920 | 1.059 |
| Year of index treatment | **2009** |  | ref. |  |  |
|  | **2010** | 0.219 | 0.959 | 0.897 | 1.025 |
|  | **2011** | 0.004 | 0.905 | 0.845 | 0.969 |
|  | **2012** | <0.001 | 0.850 | 0.791 | 0.913 |
|  | **2013** | <0.001 | 0.850 | 0.789 | 0.915 |
|  | **2014** | <0.001 | 0.817 | 0.757 | 0.882 |
|  | **2015** | <0.001 | 0.852 | 0.785 | 0.924 |
|  | **2016** | <0.001 | 0.841 | 0.770 | 0.918 |
|  | **2017** | <0.001 | 0.803 | 0.722 | 0.894 |

c: complicated, uc: uncomplicated; for the yes/no variables, the hazard ratio for yes vs. no is shown

**Table S4** Adjusted hazard ratios for overall survival (SHI) according to continuity of certification (treatment in centers)

| Continuity of certification | p-value | HR* | lower CI | upper CI |
| --- | --- | --- | --- | --- |
| not certified |  | ref. |  |  |
| <1 year | 0.004 | 0.859 | 0.775 | 0.953 |
| 1-<2 years | 0.602 | 0.972 | 0.872 | 1.083 |
| 2-<5 years | 0.008 | 0.887 | 0.812 | 0.968 |
| 5 or more years | 0.000 | 0.765 | 0.672 | 0.870 |

* Multivariable Cox regression - adjusted for age, year of diagnosis, elixhauser comorbidities and hospital characteristics

**Table S5** Full results for adjusted multivariable Cox regression for overall survival following treatment in centers (CCR)

|  |  | ***p*-value** | **HR** | **lower 95% CI** | **upper 95% CI** |
| --- | --- | --- | --- | --- | --- |
| **Treatment in center** | **no** |  | ref. |  |  |
|  | **yes** | 0.490 | 0.964 | 0.867 | 1.071 |
| **Age at diagnosis (years)** | **0–49** |  | ref. |  |  |
|  | **50–59** | <0.001 | 1.468 | 1.193 | 1.807 |
|  | **60–69** | <0.001 | 1.983 | 1.633 | 2.409 |
|  | **70–79** | <0.001 | 2.964 | 2.459 | 3.573 |
|  | **80+** | <0.001 | 5.671 | 4.653 | 6.911 |
| **Year of diagnosis** | **2009–2011** |  | ref. |  |  |
|  | **2012–2014** | 0.253 | 1.058 | 0.960 | 1.166 |
|  | **2015–2017** | 0.248 | 1.078 | 0.949 | 1.224 |
| **Stage** | **I** |  | ref. |  |  |
|  | **II** | 0.146 | 1.256 | 0.924 | 1.706 |
|  | **III** | <0.001 | 2.963 | 2.435 | 3.606 |
|  | **IV** | <0.001 | 4.449 | 3.637 | 5.443 |
|  | **X/n.s.** | <0.001 | 3.580 | 2.901 | 4.420 |
| **Grade** | **G1** |  | ref. |  |  |
|  | **G2** | 0.005 | 1.614 | 1.160 | 2.246 |
|  | **G3/4** | 0.001 | 1.713 | 1.238 | 2.369 |
|  | **GX/n.s.** | <0.001 | 1.975 | 1.418 | 2.750 |
| **Lymphatic invasion** | **L0** |  | ref. |  |  |
|  | **L1** | <0.001 | 1.281 | 1.114 | 1.473 |
|  | **LX/n.s.** | 0.006 | 1.519 | 1.129 | 2.046 |
| **Venous invasion** | **V0** |  | ref. |  |  |
|  | **V1/2** | 0.291 | 1.085 | 0.933 | 1.261 |
|  | **VX/n.s.** | 0.259 | 1.174 | 0.889 | 1.550 |

n.s.: not specified

**Table S6** Hazard ratios for recurrence-free survival (CCR) following treatment in centers

| **Variable** | **Category** | **Univariable Cox regression** | | | | **Multivariable* Cox regression** | | | |
| --- | --- | --- | --- | --- | --- | --- | --- | --- | --- |
|  |  | ***p*-value** | **HR** | **lower CI** | **upper CI** | ***p*-value** | **HR** | **lower CI** | **upper CI** |
| **Treatment in center** | No |  | 1.000 |  |  |  | 1.000 |  |  |
|  | Yes | 0.304 | 0.896 | 0.727 | 1.105 | 0.379 | 0.906 | 0.727 | 1.129 |

* adjusted for age at diagnosis, year of diagnosis, stage UICC, grading, lymphatic invasion, vein invasion

*p* = log-rank *p*-value, HR = hazard ratio, CI = confidence interval, X = not determinable, ns not specified

*p* in the line of reference category denotes *p*-value for the variable‘s total effect
